# The complex character of photosynthesis in cucumber fruit

**DOI:** 10.1093/jxb/erx034

**Published:** 2017-03-27

**Authors:** Xiaolei Sui, Nan Shan, Liping Hu, Cankui Zhang, Changqing Yu, Huazhong Ren, Robert Turgeon, Zhenxian Zhang

**Affiliations:** 1 Beijing Key Laboratory of Growth and Developmental Regulation for Protected Vegetable Crops, College of Horticulture, China Agricultural University, Beijing 100193, China; 2 Beijing Vegetable Research Center, Beijing Academy of Agriculture and Forestry Sciences, Beijing 100097, China; 3 Department of Agronomy, Purdue University, West Lafayette, IN 47907, USA; 4 Ecological Laboratory, Ecotech Ecological Technology Ltd, Beijing 100190, China; 5 Plant Biology Section, Cornell University, Ithaca, NY 14853, USA

**Keywords:** Chloroplast, cucumber, fruit photosynthesis, PEPC (phosphoenolpyruvate carboxylase), respiration, Rubisco (ribulose-1,5-bisphosphate carboxylase/oxygenase).

## Abstract

The surface area of a mature green cucumber (*Cucumis sativa* L.) fruit is comparable with that of a functional leaf, but the characteristics of fruit photosynthesis and its contribution to growth are poorly understood. Here, the photosynthetic properties of two genotypes of cucumber (dark green and light green fruits) were studied using a combination of electron microscopy, immunogold enzyme localization, chlorophyll fluorescence imaging, isotope tracer, and fruit darkening techniques. Chlorophyll content of the exocarp is similar to that of leaves, but there are no distinctive palisade and spongy tissues. The efficiency of PSII is similar to that in leaves, but with lower non-photochemical quenching (NPQ). Ribulose-1,5-bisphosphate carboxylase/oxygenase (Rubisco) is found mainly in the exocarp, while phosphoenolpyruvate carboxylase (PEPC) is primarily localized to vascular bundles and placenta tissue. Rubisco and PEPC expression at both transcriptional and translational levels increases concurrently during fruit growth. The contribution of fruit photosynthesis in exocarp to its own C accumulation is 9.4%, while ~88% of respiratory CO_2_ in fruit was captured and re-fixed. Photosynthesis by cucumber fruits, through direct fixation of atmospheric CO_2_ and recapture of respired CO_2_, as verified by ^14^CO_2_ uptake and gas exchange, makes an important contribution to fruit growth.

## Highlight

Photosynthesis by cucumber fruits, through direct fixation of atmospheric CO_2_ and recapture of respired CO_2_, makes an important contribution to fruit growth.

## Introduction

Leaves are not the only organs that conduct photosynthesis. A variety of chlorophyll (Chl)-containing non-foliar vegetative and reproductive organs produce photoassimilates; for example, celery petioles and tobacco stems (Hibberd and Quick, 2002), sink organs including cotton bolls (Hu *et al.*, 2012), immature tomato fruits (Xu *et al.*, 1997; Hetherington *et al.*, 1998; Carrara *et al.*, 2001), and even fruit subsidiary tissues including wheat awns (Li *et al.*, 2006) and maize husks (Pengelly *et al.*, 2011).

 There are two potential sources of CO_2_ for non-foliar photosynthesis: the atmosphere and the internal environment where CO_2_ is released by respiration. Effective internal re-fixation of CO_2_ released from respiratory reactions is common (Aschan and Pfanz, 2003), for example in the mid-veins of Arabidopsis leaves where photosynthetic Chl-rich cells in and around the vascular bundles possess high activities of decarboxylation enzymes that release CO_2_ from C_4_ organic acids present in the xylem transpiration stream (Brown *et al.*, 2010).

 The degree to which photosynthesis by fruits contributes to their own growth is species specific. Stomata are common on the surface of many fruits, but the number is fixed early in development so that as the fruit grows they become less frequent. Stomata on a mature apple fruit, for example, are 30 times more scarce than on the abaxial surface of an apple leaf (Blanke and Lenz, 1989). A number of studies in this field have been conducted on tomato, a model for fleshy fruits (Seymour *et al.*, 2013), in which photosynthesis is restricted to the early stages of fruit maturation when they are green.

Cucumber (*Cucumis sativus* L.) represents a fundamentally different type of model fruit that remains green throughout development and in which the surface area at maturity is almost equivalent to the area of a fully expanded leaf. Stomata are sufficiently frequent on the surface of mature fruit that some varieties are wrapped in plastic prior to sale to avoid water loss. At the same time, cucumber fruits are non-climacteric and maintain a high respiratory rate when mature. This suggests that photosynthetic capture of CO_2_, both from the atmosphere and from respiration, contributes significantly to fruit growth. If so, it is likely that a complex co-ordination of photosynthetic pathways occurs concurrently in the same, or in closely positioned tissues.

At present, little is known about fruit photosynthesis in cucumber, including such important parameters as the ratio of atmospheric and respiratory sources of CO_2_ and the relative importance of Rubisco (ribulose-1,5-bisphosphate carboxylase/oxygenase; EC 4.1.1.39) and PEPC (phosphoenolpyruvate carboxylase; EC 4.1.1.31) in fixation. We used a variety of techniques to better understand C balance and other aspects of fruit photosynthesis in this model species.

## Materials and methods

### Plant materials, growth conditions, and treatments

Two cucumber genotypes ‘Zhongnong No.16’ (‘ZN16’, from the Institute of Vegetables and Flowers, Chinese Academy of Agricultural Sciences) and ‘Baiyesan’ (‘BYS’) were pre-cultured in a phytotron with a 10 h photoperiod and a temperature cycle of 25/18 ºC (day/night). The photon flux density (PFD) was 500 µmol m^–2^ s^–1^. Seedlings were transferred to a solar greenhouse and arranged in a completely randomized design with three replicates. Normal cucumber fruits were collected at –2, 0, 3, 6, and 9 days after anthesis (DAA). In the same experiment, fruits were bagged/darkened with a double-layer black kraft paper bag (0.0804 mm thick, density 58.76 g m^–2^) to exclude light and were harvested 9 days after darkening (DAD) on the day of flowering. At the same time, leaves of ‘ZN16’ and ‘BYS’ at 9 days after unfolding (DAU) were sampled to analyze the same parameters as for fruits. Unless stated otherwise, all chemicals and enzymes were purchased from Sigma-Aldrich (USA).

### Light and electron microscopy

For light microscopy, free-hand transverse sections were prepared for stereoscopic (Leica S8 APO) observation. For paraffin sectioning, fruit tissues were cut into small pieces (maximum of 5 × 5 mm) and immediately fixed at 4 °C for 24 h in FAA [70% (v/v) ethanol, 5% (v/v) acetic acid, and 2% (v/v) formalin]. After dehydration through an ethanol gradient series, tissues were embedded in Paraplast Plus (Fisher, USA), sectioned at a thickness of 8–10 μm, then stained in safranin and fast green. For semi-thin sectioning (0.5–1.0 μm), tissues were fixed in glutaraldehyde at 4 °C for 24 h, post-fixed in osmium tetroxide, dehydrated in a graded acetone series, then infiltrated and embedded with Spurr resin. Images were obtained using an optical microscope (Olympus BX53). For TEM, the ultrathin sections from Spurr-embedded blocks were stained with lead citrate and uranyl acetate, and viewed with a HITACHI-7500 transmission electron microscope (Zhang *et al.*, 2004). The number of chloroplasts per unit area was calculated with >10 different microscope fields for each treatment. Sections of leaf surfaces (both upper and lower epidermis) and fruits (exocarp) were dissected for SEM observation. Samples were fixed in glutaraldehyde, dehydrated in ethanol, critical-point dried, and gold coated. Stomata were observed with a HITACHI S-3400 scanning electron microscope. Stomatal frequency was calculated in 50 different fields of view (0.025 mm^2^×50) per sample.

### Determination of surface area, and contents of pigments and organic acids

Total leaf surface area was estimated by tracing leaves onto uniform-weight paper and weighing the cut-outs. The surface area of fruits was estimated as described previously, approximating them as cylinders (Marcelis and Baan Hofman-Eijer, 1995). Fifteen random samples were used at each fruit developmental stage. Chl content was measured according to standard methods (Lichtenthaler, 1987; Porra *et al.*, 1989). Total organic acids were determined using the acid–base titration method (Hoffmann *et al.*, 1989).

### Quantitative real-time PCR analysis

Total RNA was extracted using a Trizol Kit (Invitrogen) according to the manufacturer’s instructions. After removal of the potential genomic DNA with RNase-free DNase I, the DNA-free RNAs were reverse transcribed (SuperScript II, Invitrogen, USA), using an oligo(dT)_18_ primer, into single-stranded cDNA which was used as the template for quantitative real-time PCR analysis. The gene-specific primers used for analysis are listed in Supplementary Table S1 at *JXB* online. PCR products were amplified in triplicate using the iQ™ SYBR-Green Supermix (Bio-Rad, USA) in 25 µl reactions. Three biological replicates were performed for each reverse transcription–PCR procedure. Threshold cycle values were calculated using iCycler software, and cucumber *tubulin* was used as internal control (Wan *et al.*, 2010). Relative expression levels were calculated according to the 2^–ΔΔCT^ method (Livak and Schmittgen, 2001).

### Enzyme assay and immunoblotting

Enzymatic activities of the Rubisco and PEPC enzymes were measured as described previously (Cousins *et al.*, 2007; Pengelly *et al.*, 2010) with some modifications. The activity of both enzymes was calculated by monitoring the decrease of NADH absorbance at 340 nm with a spectrophotometer (Unico UV-2802PC, USA).

 Immunoblotting was performed according to Cousins *et al.* (2007) with some modifications. After SDS–PAGE, the gels were subjected to immunoblotting using antisera including anti-Rubisco large subunit-RBCL (Agrisera, Sweden, product no. AS03 037), anti-Rubisco small subunit RBCS (Agrisera product no. AS07 259), or anti-PEPC (Agrisera product no. AS09 458) of *Arabidopsis thaliana*. The total soluble protein content was determined according to Bradford (1976).

### *In situ* hybridization

Pre-treatment of samples (fixing, dehydration and embedding) was similar to that used in paraffin sectioning. Cross-sections (10 μm thick) were sliced and mounted on ProbeOn Plus Slides (Fisher). Sense and antisense riboprobes were synthesized by *in vitro* transcription from PCR products under the SP6 or T7 promoter with RNA polymerase using the DIG RNA labeling kit (Roche, USA). Primer sequences are listed in Supplementary Table S1. The selectivity of the AgMaT1 probe was checked through a dot-blot experiments. *In situ* hybridization was performed according to Jackson (1991). Images were obtained using an Olympus BX53 microscope. For each gene-specific probe analyzed, at least three replicate samples were hybridized.

### Immunolocalization

Immunolocalization of Rubisco and PEPC was performed as described by Wang *et al.* (2009). Briefly, sample pre-treatment including fixation, dehydration, embedding, and section cutting was done using the same method as for *in situ* hybridization. The sections were then blocked, incubated first in the primary RBCL (diluted 1:500) and PEPC (diluted 1:200) antibody, respectively, and subsequently incubated with a 1:1000 dilution of secondary antibody [goat anti-rabbit IgG–fluorescein isothiocyanate (FITC), Jackson, USA, diluted 1:200]. Images were obtained under an Olympus fluoview FV1000 confocal laser scanning microscope with 488 nm excitation wavelength, and chloroplast fluorescence was observed at a 546 nm wavelength.

### Immunogold localization

The method was adapted from Zhang *et al.* (2004) and Pengelly *et al.* (2011) with modifications. Samples after fixation, as above, were embedded in LR White acrylic resin (London Resin Co., Basingstoke, UK). Ultrathin sections (60–90 nm) were made and then mounted on 100 mesh nickel grids coated with 0.3% formvar films. After blocking, the ultrathin sections were incubated with primary antibodies [anti-RBCL (1:1000) or anti-PEPC (1:50)] and secondary antibodies (goat anti-rabbit IgG antibody conjugated with 10 nm gold). Following extensive washes, the sections were treated by silver-enhanced staining and post-stained with 2% uranyl acetate. Images were obtained with a HITACHI-7500 transmission electron microscope.

### Chlorophyll fluorescence imaging

Chl fluorescence images were taken using an imaging fluorometer (FluorCam 1000-H, P.S.Instruments, Brno, Czech Republic). Plant material was first dark-adapted for 30 min at room temperature. A laboratory-built pneumatic shutter system provided 800 ms saturating light pulses (2000 μmol m^–2^ s^–1^ PFD) during a 1 min illumination with actinic light (500 μmol m^–2^ s^–1^ PFD). The opening and closing of the shutters were controlled by a laboratory-built digital interface connected to a personal computer. Color video images of *F*_m_ (maximum fluorescence in the dark-adapted state), *F*_m_' (steady-state maximum fluorescence in light), or *F*_t_ (steady-state fluorescence in light) were captured, and false-color images of maximum PSII quantum yield (*F*_v_/*F*_m_), steady-state PSII quantum yield (ΦPSII), and steady-state non-photochemical quenching (NPQ) were generated as described previously (Genty *et al.*, 1989; Horton and Ruban, 1992).

### Gas exchange analysis

Gas exchange measurements were performed using a LI-6400 portable photosynthesis system (Li-Cor, Inc., Lincoln, NE, USA) equipped with an infrared gas analyzer (IRGA, 6400-02B). Cucumber plants were first adapted to gas exchange light intensity conditions (usually 1 h prior to the measurement), and then leaves attached to the stem or fruit exocarp samples were prepared and immediately put into the leaf chamber. CO_2_ assimilation/evolution rates were measured under various light intensities as well as in dark-adapted leaves and fruits. CO_2_ concentration, air temperature, relative humidity, and air flow rate inside the leaf chamber were maintained automatically at 400 ± 10 μmol mol^–1^, 28 ± 1 ^o^C, 50–60%, and 500 μmol s^–1^, respectively.

In order to calculate carbon balance in the marketable mature fruit (9 DAA), the CO_2_ evolution rates of intact fruits (R_I_/R_I_'), peels (~150–200 μm thickness) (R_P_/R_P_'), and slices without peels (R_S_/R_S_') were measured in the light and dark using a plexiglas fruit chamber, equipped with a fan and a CO_2_ gas sensor system (Vernier Software & Technology, LLC, Beaverton, OR, USA). Peels and slices were put onto wet plastic tape to prevent CO_2_ exchange at the interface of tissue and tape. Wound respiration of slices caused by cutting, which is defined as R_W_, was corrected for when R_S_ or R_S_' was determined. The fruit (without peel) were cut into slices of different thickness: 1, 2, 3, 4, and 5 mm. Then CO_2_ evolution of those slices was measured and the computational one-dimensional linear regression equation was obtained (see Supplementary Fig. S12). The intercept on the y-axis is approximately equal to wounded respiration of slices, which was calibrated when R_S_ or R_S_' is determined. The net photosynthetic rate of sampled tissues was defined as the difference between the CO_2_ evolution rates in the light and the dark. Relative recaptured CO_2_ in fruit in the light (%) is [(R_S_+R_P_–R_I_)/(R_S_+R_P_)]×100, and in the dark is [(R_S_'+R_P_'–R_I_')/(R_S_'+R_P_')]×100.

### ^14^CO_2_ feeding and autoradiography

^14^CO_2_ feeding was conducted at 09.00 h on a sunny day using a protocol modified from Kanai *et al.* (2007). Fruits were enclosed in polypropylene plastic bags and the radiolabel was injected into a vial inside the bag. Each fruit received 3.7 × 10^6^ Bq of ^14^CO_2_ derived from [^14^C]Na_2_CO_3_, and was allowed to assimilate ^14^CO_2_ for 1.5 h under natural light conditions. Lower fruits that had been darkened for 6 d after anthesis (6 DAD) were treated with ^14^CO_2_ after being exposed to natural light in the greenhouse for 1.5 h, then darkened again by bagging. Those plants were harvested 24 h after ^14^CO_2_ treatment. The specific activity of ^14^C in each sample was analyzed by an LS 6500 Multi-Purpose scintillation counter (Beckman Coulter, Inc., USA). After ^14^CO_2_ labeling, fruit tissue for autoradiography was selected and sectioned, and autoradiography was performed as described previously (Zhang *et al.*, 2004).

### Fruit bagging and the relative contribution to fruit yield

At the 10- to 12-leaf stage, plants were pruned to different leaf-to-fruit ratios [i.e. keeping eight leaves/two fruits, four leaves/two fruits (control), and two leaves/two fruits on a plant] (Supplementary Fig. S1). For each leaf-to-fruit ratio treatment, either the upper fruit, both fruits, or no fruits (control) were bagged/darkened with black kraft paper at 0 DAA to exclude light. Upper or lower fruits on the same plant were collected separately, and weighed at the marketable mature stage (9–10 DAA). For each darkening treatment under different leaf-to-fruit ratios, 20 replicate samples/plants were analyzed. The relative contribution of fruit photosynthesis to carbon requirement in fruit yield was calculated as follows (Hu *et al.*, 2012): Relative contribution (%) = [(control yield–yield in darkness of bagged fruits)/control yield]×100. In addition, the dry weights of leaves and stems of ‘ZN16’ were measured after harvest.

### Data analysis

All data were subjected to ANOVA using SPSS statistical software version 17.0. The data were presented as the mean ±SD. The significance of differences between mean values was determined with Tukey’s test at *P*<0.05.

## Results

### Ultrastructure of exocarp and chloroplasts

**Fig. 1.**
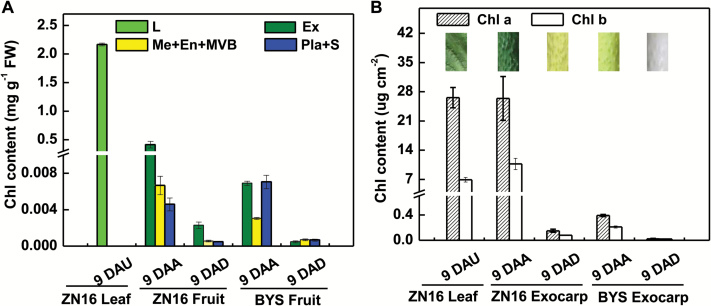
Chlorophyll content in fruits and leaves based on per unit fresh weight (A) and surface area (B). Images in (B) were obtained from a leaf at 9 DAU and a fruit at 9 DAA/9 DAD, respectively, illustrated by different colors. Error bars represent the SD, *n*=3. DAA, days after anthesis; DAD, days after darkening; DAU, days after unfolding; Chl, chlorophyll; En, endocarp; Ex, exocarp; L, leaf; Me, mesocarp; Pla, placenta; S, seeds; MVB, main vascular bundle.

To explore the photosynthetic characteristics of cucumber fruit, two genotypes, one dark green (ZN16) and one light green (BYS) at maturity (Supplementary Fig. S2A), were studied. Pigment analysis confirmed that Chl mainly resides in the exocarp and that the Chl content in ‘ZN16’ exocarp is much higher than in ‘BYS’ (Fig. 1; Supplementary Fig. S2C), and when calculated on a per unit area basis is comparable with that of mature leaves (Fig. 1B). In particular, the exocarp parenchyma cells contain a relatively high content of Chl *b* (Fig. 1B) but a lower Chl *a*/*b* ratio (1–2.5:1) than that in the leaf (3–4:1), which is associated with adaptation to low light intensity.

**Fig. 2.**
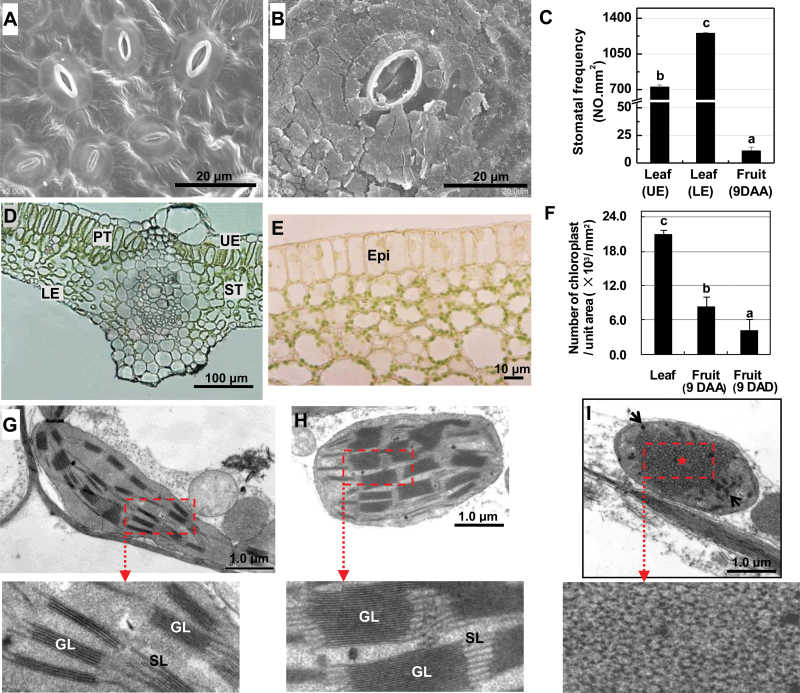
Anatomical features of chloroplasts of cucumber exocarp. (A, B) Scanning electron micrographs of the lower epidermis of a mature leaf (9 DAU) (A) and epidermis of fruit (9 DAA) (B). The stomata are covered with cuticular wax on the fruit epidermis. (C) Stomatal frequency. (D, E) Cross-section of a leaf at 9 DAU (D) and a fruit at 9 DAA (E). (F) Number of chloroplasts per unit area. (G–I) Transmission electron micrographs of chloroplasts distributed in palisade cells of a mature leaf (9 DAU) (G), parenchyma cells of fruit (9 DAA) (H), and darkened fruit (9 DAB) (I), respectively. Variety: ‘ZN16’. Means followed by different letters indicate statistically significant differences according to Tukey’s test (*P*<0.05) [(*n*=50 in (C); *n*=10 in (F)]. Epi, epidermis; GL, grana lamellae; LE, lower epidermis; PT, palisade tissue; SL, stroma lamella; ST, spongy tissue; UE, upper epidermis.

 ‘ZN16’ fruit grows rapidly after anthesis and the superficial area at maturity (9 DAA) is ~230 cm^2^, which is comparable with the surface area of a single growing leaf at the same time period (Supplementary Fig. S2A, B). Stomata are significantly larger on the ‘ZN16’ fruit surface (Fig. 2B) than those on leaves (Fig. 2A). However, stomatal frequency is only 1.58% and 0.91% that of the upper and lower surfaces of leaves, respectively (Fig. 2C). These percentages are similar to those in other species with photosynthetic fruits (only 1–10%; Blanke and Lenz, 1989). At 9 DAA, ~10 layers of parenchyma cells form a tissue 160–170 μm in depth under the fruit epidermis, which is equivalent to the thickness of mature leaves. Unlike leaves (Fig. 2D), there are no palisade and spongy tissue structures in cucumber fruits, and intercellular spaces are very small (Fig. 2E). A large number of chloroplasts appear on the inner walls of fleshy parenchyma cells under the epidermis (Fig. 2E), but the quantity per unit area in these fruit tissues is still much lower than in leaves (Fig. 2F). The size of the grana stacks of fruit chloroplasts (Fig. 2H) is 1.7-fold larger than in leaves (Fig. 2G), resembling the chloroplasts of shade plants (Lichtenthaler and Burkart, 1999).

 When darkened by bagging for 9 DAA, the exocarp of ‘ZN16’ fruits were dark green to yellowish green, and darkened ‘BYS’ fruits were white rather than light green (Fig. 1; Supplementary Fig. S2A). Light exclusion during growth also resulted in the development of very few chloroplasts in the ‘ZN16’ exocarp (Fig. 2F). Moreover, reticular prolammellar bodies with clear plastoglobuli, but no distinct grana stacking, were observed in chloroplasts/etioplasts in the exocarp of darkened fruit at 9 DAB (Fig. 2I). Because well-developed chloroplasts had been formed by the day of flowering (data not shown), these data indicate that darkening during development significantly induces decomposition of pigment (Chl) and degradation of developed chloroplasts and thylakoid membranes.

### Gene expression and enzymatic activity of Rubisco and PEPC

**Fig. 3.**
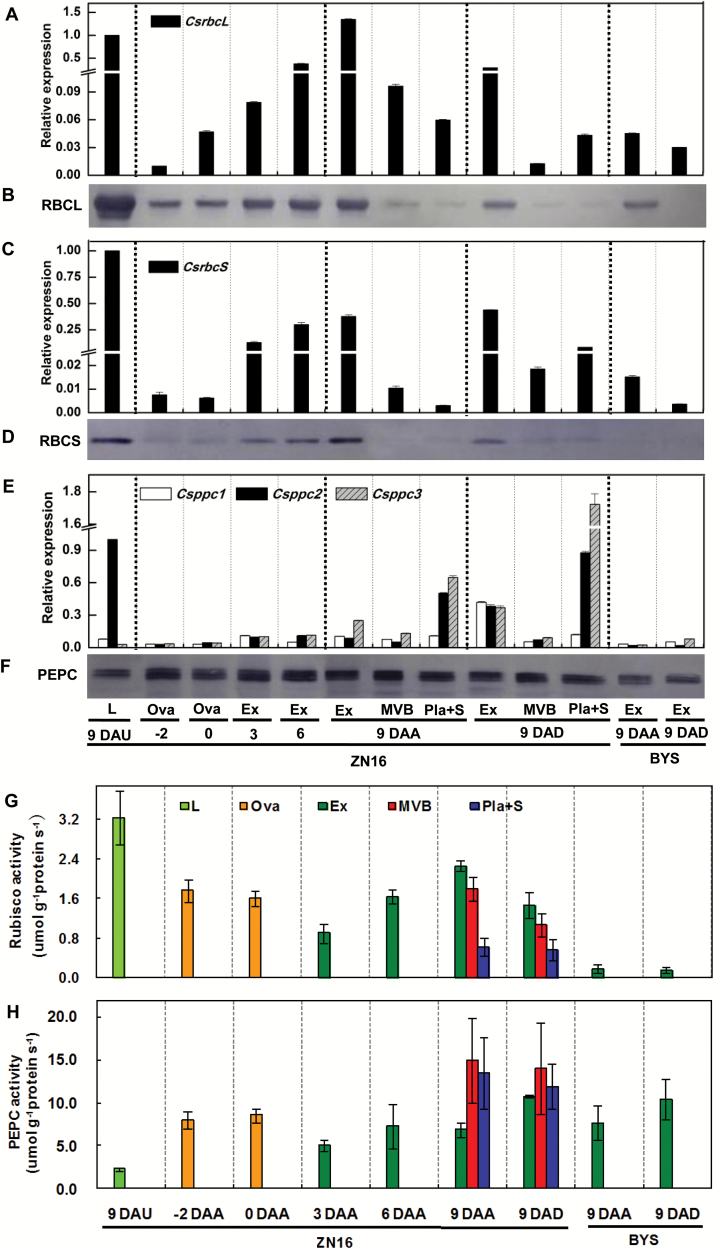
Expression and activities of Rubisco and PEPC in cucumber fruits. (A–F) Quantitative real-time PCR analysis of *rbc*L (A), *rbc*S (C), and *ppc* (E) mRNA levels, and immunoblot analysis of the Rubisco large (RBCL) (B) and small (RBCS) (D) subunits and PEPC (F) using antibodies to each of the respective proteins. Experiments for the quantification of protein levels were repeated three times, yielding similar results. (G, H) Enzymatic activities of Rubisco (G) and PEPC (H), which were calculated per protein concentration. Error bars represent the SD, *n*=3. For abbreviations, see Fig. 1.

Quantitative real-time PCR and immunoblotting assays indicated that *rbc*L and *rbc*S, encoding the large and small subunits of Rubisco, respectively, and *ppc* families, encoding PEPC, were ubiquitously expressed at the transcriptional and translational levels in all the sampled parts of ‘ZN16’ fruits and leaves, and their expression level in fruit increased as fruit developed and reached the maximum level at the marketable mature stage (9 DAA) (Fig. 3A–F). Among three members of the *ppc* gene family (http://cucumber.genomics.org.cn/), *Csppc2* was expressed mainly in leaves and at a relatively lower level in the fruits, while *Csppc3* was expressed mainly inside fruits, especially in placenta and seed. The activities of the Rubisco holoenzyme (Fig. 3G) and PEPC (Fig. 3H) increased in proportion to transcript abundance (Fig. 3A, C, E) during fruit development. However, gene expression of *rbc*L and *rbc*S (Fig. 3A–D) and enzyme activity of Rubisco (Fig. 3G) were mainly in leaves and exocarp. In contrast, *Csppc* expression (Fig. 3E, F) and PEPC activity (Fig. 3H) in the internal parts [main vascular bundle (MVB) and placenta tissue] of 9 DAA fruit were much higher than in the exocarp, and were also significantly higher than in leaves of a similar age (9 DAU). Similar results for expression (Supplementary Fig. S3A, B) and activities (Supplementary Fig. S3C) of Rubisco and PEPC in leaves and developing fruits of cucumber ‘BYS’ were also obtained. However, in ‘BYS’, the abundance levels of *rbc*L, *rbc*S, and *Csppc* mRNA (Fig. 3A, C, E) and polypeptide proteins (Fig. 3B, D, F) were lower than each of the respective genes in ‘ZN16’.

 Total organic acid analysis of cucumber fruit indicated that high levels of organic acid were present in fruit compared with leaf, especially during rapid growth (Supplementary Figs S2, S4). Presumably, organic acid in fruits is synthesized through the PEPC pathway.

Darkening by bagging significantly repressed *rbc*L mRNA expression (Fig. 3A) and Rubisco holoenzyme activity (Fig. 3G) in the exocarp. In contrast, darkening substantially increased the expression level of *Csppc* in fruits (Fig. 3E), but had no significant effect on the corresponding PEPC enzyme activity in either ‘BYS’ or ‘ZN16’ fruits (Fig. 3H). In addition, either with or without darkening, PEPC activity in fruit was much higher than Rubisco activity, in particular 10–20 times in the perivascular and placenta tissues (Fig. 3G, H).

### Histochemical and subcellular localization of Rubisco and PEPC

**Fig. 4.**
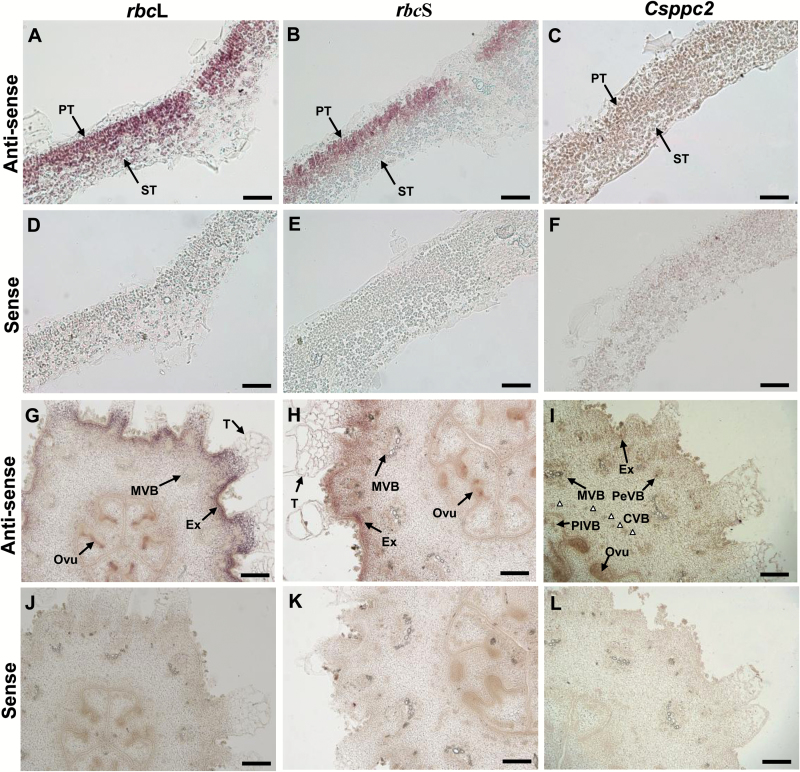
*In situ* hybridization of *rbc*L, *rbc*S, and *ppc* transcripts in cucumber fruits. (A–F) Leaf cross-sections (0–1 DAU) hybridized with the *rbc*L, *rbc*S, and *Csppc2* antisense (A–C) and sense (D–F) probes, respectively. (G–L) Young ovary/fruit cross-sections (–2 –0 DAA) hybridized with the *rbc*L, *rbc*S, and *Csppc2* antisense (G–I) and sense (J–L) probes, respectively. White triangles in (I) indicate the CVB. CVB, carpel vascular bundle; Ovu, ovule; PeVB, peripheral vascular bundle; PlVB, placenta vascular bundle; T, trichome; for other abbreviations, see Figs 1 and 2. Scale bars=50 µm in (A–F) and 200 µm in (G–L).

**Fig. 5.**
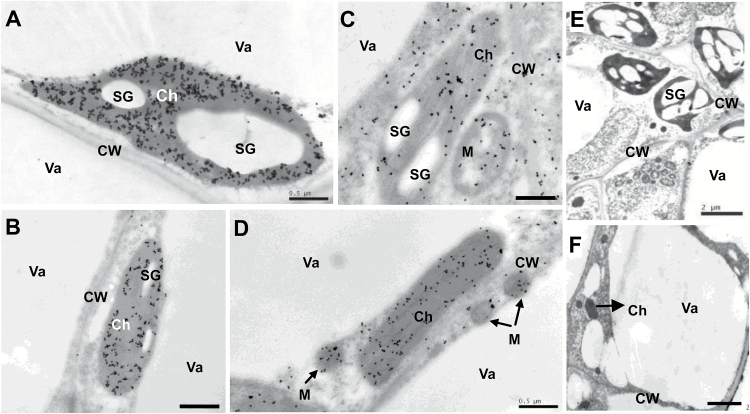
Immunogold localization of Rubisco and PEPC in cucumber fruits. (A, B) Rubisco (gold particles) was localized to chloroplasts in leaf (A) and ovary/fruit (B). (C, D) PEPC was targeted to the chloroplast, cytoplasm, and mitochondria in leaf (C) and ovary/fruit (D). (E, F) The controls of leaf (E) and ovary/fruit (F) without incubation with antibodies; no substantial signals were detected. Sections were prepared from fully expanded leaves and ovaries/fruits on the day of anthesis. Scale bars=0.5 µm in (A–D), and 2 µm in (E, F). Ch, chloroplast; CW, cell wall; M, mitochondrion; SG, starch grain; Va, vacuole.

*In situ* hybridization results indicated that Rubisco genes are expressed mainly in palisade tissues of leaves (Fig. 4A, B, D, E) and in the exocarp, and at a low level in MVB, placenta tissue, trichomes, and parenchyma (Fig. 4G, H, J, K), while the *Csppc2* gene is expressed in low levels in all parts of leaves (Fig. 4C, F), but at a high level in the inner tissue of the fruit, especially placenta tissue and vascular bundles, and to a limited degree in the exocarp (Fig. 4I, L). Localization patterns of Rubisco and PEPC protein (Supplementary Fig. S5) were similar to those of their respective transcripts (Fig. 4). Furthermore, immunogold localization analysis indicated that Rubisco is mainly targeted to chloroplasts (Fig. 5A, B), while PEPC in cucumber fruits and leaves is found in chloroplasts, cytoplasm, and mitochondria (Fig. 5C, D). Very few, randomly placed, gold particles were observed in the control experiments (Fig. 5E, F), which confirmed that the antibodies are highly specific.

### Chl fluorescence imaging

**Fig. 6.**
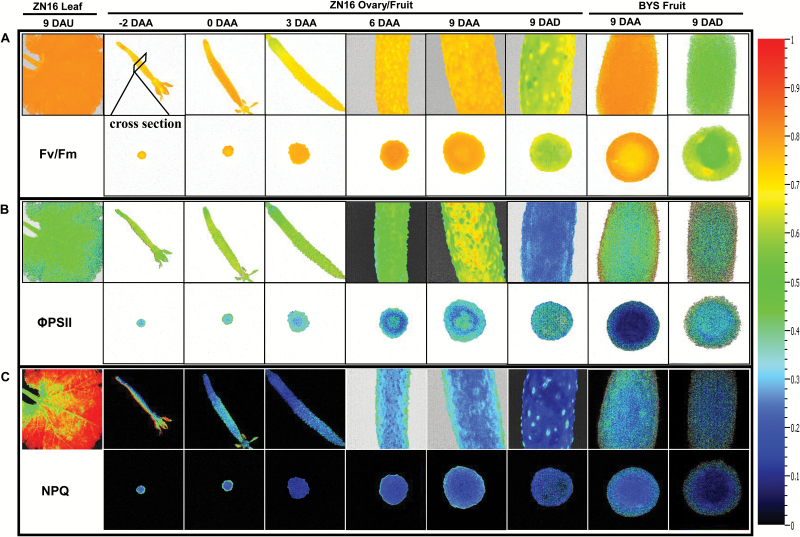
Chlorophyll fluorescence imaging of cucumber fruits. (A–C) Fluorescence images correspond to *F*_v_/*F*_m_ (A), ΦPSII (B), and NPQ (C). The upper row shows images of the whole or partial fruits; in the lower row are the corresponding cross-sections. All images were normalized to a false color bar (see right column). The pixel value display is based on a false-color scale ranging from blue (0.0), green, yellow, to red (ending at 1.0). The analyses of *F*_v_/*F*_m_ were carried out on dark-adapted fruits and leaves, while those of ΦPSII and NPQ were at a light intensity of 500 μmol quanta m^–2^ s^–1^. *F*_v_/*F*_m_, maximum PSII quantum yield; ΦPSII, steady-state PSII quantum yield; NPQ, steady-state non-photochemical quenching.

Chl fluorescence imaging identifies spatial heterogeneities of photochemical reactions in live tissues (Baker, 2008). PSII photochemical efficiencies (*F*_v_/*F*_m_ and ΦPSII) during fruit development (from –2 DAA to 9 DAA) were both high in the exocarp, and almost the same as in leaves (Fig. 6A, B). These results indicated that the photosynthetic machinery for the absorption and conversion of light energy is established rapidly when fruit is very young. NPQ in cucumber fruits did not change markedly during fruit development, and was significantly lower than in leaves (Fig. 6C). Generally, in addition to a slightly lower ΦPSII of the inner fruit tissues than of the exocarp (Fig. 6B), there was no significant spatial heterogeneity in photochemical reaction activity for cucumber fruits at the mature stage (Fig. 6; Supplementary Fig. S6). Fruit darkening treatments led to a significant reduction in *F*_v_/*F*_m_ (Fig. 6A), ΦPSII (Fig. 6B), and NPQ (Fig. 6C). ΦPSII and NPQ in ‘BYS’ were slightly lower than in ‘ZN16’ (Fig. 6B, C), whereas there was no significant difference in *F*_v_/*F*_m_ (Fig. 6A) between the two cultivars.

### CO_2_ assimilation and allocation

**Fig. 7.**
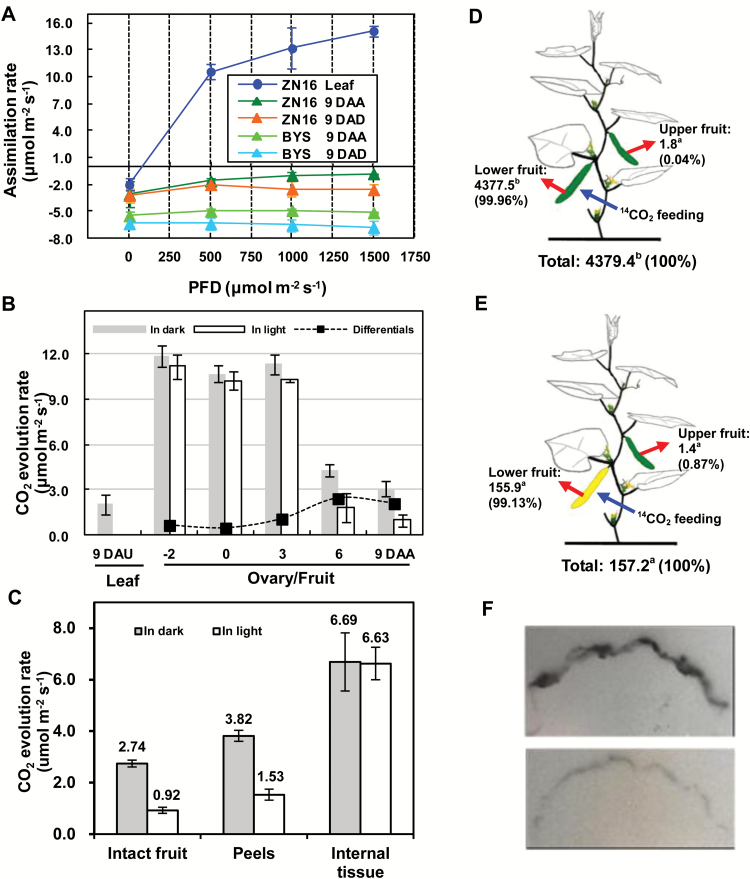
CO_2_ assimilation and ^14^C feeding in cucumber fruits. (A) The response curve of assimilation rate to photon flux density (PFD). (B) CO_2_ evolution rate of exocarp (peels) in the dark and illuminated (1000 µmol quanta m^–2^ s^–1^ irradiance) conditions during ‘ZN16’ fruit development. The net photosynthetic rate per unit fruit surface area (black squares) was the difference between the CO_2_ evolution rates in the light and dark. All data above were determined at ambient CO_2_ between 390 mbar and 410 mbar and at air temperature of 28°C. (C) CO_2_ evolution rate of intact fruit, peels, and internal tissue under dark and light (1000 µmol quanta m^–2^ s^–1^ irradiance) conditions, respectively (Variety ‘ZN16’, 9 DAA). Error bars represent the SD, *n*=3. (D, E) Allocation of ^14^C in cucumber fruits. Lower non-bagged (D) or bagged (E) fruits were fed with 3.7 × 10^6^ Bq ^14^CO_2_ (blue arrows). After 24 h, specific activities (Bq g^–1^ DW) of lower and upper fruits (red arrows) were determined. The means (*n*=3) followed by different letters indicate statistically significant differences according to Tukey’s test (*P*<0.05). Values in parentheses are percentages of the total measurements. (F) ^14^C autoradiograph of non-darkened fruit (upper photo) and darkened fruit (lower photo) at 1 h after the ending of ^14^CO_2_ feeding.

The analysis described above demonstrates that cucumber fruits and leaves share the same or similar properties in many aspects of photosynthesis. How does the CO_2_ assimilation ability of fruits compare with that of leaves? Gas exchange analysis indicated that unlike the leaves, net photosynthesis could not be detected in cucumber fruits (Fig. 7A), due to their high rate of respiration (Fig. 7B). However, compared with in the dark, under strong light, CO_2_ evolution decreased (Fig. 7B), consistent with increased photosynthetic activity. Based on this criterion, the net photosynthetic rate at ‘ZN16’ fruit development from 6 DAA to 9 DAA was 2.1–2.4 µmol m^–2^ s^–1^ (Fig. 7B), which equals 13.8–15.8% of the leaf net photosynthetic rate per unit area (Fig. 7A). Similar results for CO_2_ evolution during ‘BSY’ fruit development and in the leaf were obtained (Supplementary Fig. S7). In addition, in ‘BSY’ fruits, CO_2_ assimilation in the exocarp of darkened and non-darkened fruits was consistently lower than in those of ‘ZN16’ under all light conditions (Fig. 7A).

 Being non-climacteric, cucumber fruits maintain a high respiration rate at maturity (Paul *et al.*, 2012). How much of the CO_2_ released in fruit is captured and re-fixed? To answer this question, peeled fruits were sliced transversely to measure CO_2_ evolution from inside the tissue, and the results were calculated on a fruit surface area basis so that the results are comparable with those from the peels and intact fruit. In the dark, CO_2_ amounts released in intact fruit, peels, and internal tissues were 2.74, 3.82, and 6.69 µmol m^–2^ s^–1^, respectively (Fig. 7C). However, in light, CO_2_ released in those three sampled parts was 0.92, 1.53, and 6.63 µmol m^–2^ s^–1^, respectively. This indicates that considerably more CO_2_ is released from inside the fruit than from peels. The amount of CO_2_ recaptured by the fruit was calculated as total respiration from internal tissues and peel minus the amount lost by whole fruit. In the dark, this value (7.77 µmol m^–2^ s^–1^) is 74% of total respiratory CO_2_, but in the light (7.24 µmol m^–2^ s^–1^) it is 88% of total respiratory CO_2_.

At 24 h after a 1.5 h ^14^CO_2_ labeling period, significant amounts of ^14^C were assimilated by cucumber fruits grown in the light (4377.5 Bq g^–1^ DW) (Fig. 7D) and less so by fruits kept in the dark by darkening (155.9 Bq g^–1^ DW) (Fig. 7E). Similar results were obtained using autoradiography (Fig. 7F; 6 DAA, upper; 6 DAD, lower). The decrease in CO_2_ assimilation ability in the exocarp of darkened fruit may be caused by damage to the chloroplast ultrastructure (Fig. 2I), decomposition of Chl (Fig. 1), and down-regulation of Rubisco in the dark (Fig. 3). Furthermore, whether the fruits were darkened or not, almost all of the ^14^C (99.13–99.96%) remained in the fed fruits; hardly any radioactively labeled carbohydrate (0.04–0.87%) was exported (Fig. 7D, E), indicating that external CO_2_ assimilated via the exocarp is primarily used for fruit growth.

### Relative contribution of fruit photosynthesis to yield

**Fig. 8.**
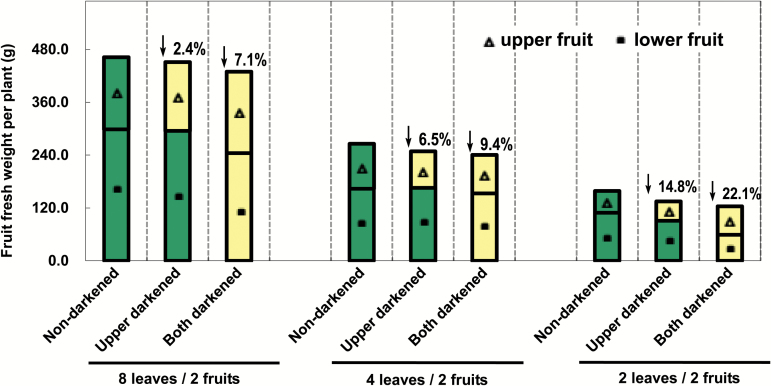
Effect of darkening cucumber fruits on fruit yield. For treatment of bagged fruits, see Supplementary Fig. S1. Values represent the average of 20 replicates. Values on the bars are the percentage decrease of fresh fruit weight after 9 d dark treatment. Green and yellow bars indicate non-darkened and darkened fruits, respectively.

To study further the contribution of fruit photosynthesis to yield, fruit darkening experiments (from 0 DAA to 9–10 DAA) were carried out on ‘ZN16’ plants with different leaf/fruit ratios (Supplementary Fig. S1). The results showed that in both darkened and non-darkened plants, fresh fruit weight per plant decreased with a decreasing leaf/fruit ratio (Fig. 8). These results indicate that the source/sink ratio is critically important for yield. Because fruits themselves also have photosynthetic capability, darkening treatments can result in lower fruit yield, and fruit yield decreased as the leaf/fruit ratio decreased, especially when both fruits were bagged. Compared with the non-darkened control plants, the two fruit-darkening treatments reduced yield by 7.1, 9.4, and 22.1% in plants with eight leaves/two fruits, four leaves/two fruits, and two leaves/two fruits, respectively (Fig. 8). Darkening the upper fruit had no effect on growth of the lower fruit (Fig. 8). In addition, fruit darkening treatments did not show any significant effect on dry matter accumulation of adjacent stems and leaves (Supplementary Fig. S8)

## Discussion

### The structure of fruit with respect to photosynthesis

Generally, photosynthesis of non-foliar tissues can be achieved by assimilation of atmospheric CO_2_ by Rubisco and/or re-fixation of released CO_2_ within tissues by PEPC, both of which are considered important strategies for additional C acquisition (Wullschleger *et al.*, 1991; Aschan and Pfanz, 2003). These conclusions were confirmed by gas exchange and ^14^C tracer analyses in this study (Fig. 7). This indicated that cucumber fruits are both sink organs and source organs. Unlike leaves, cucumber fruit is thick, cylindrical in shape, and lacks differentiated palisade and spongy tissues, but fleshy parenchyma cells, especially those embedded in the exocarp, contain comparable Chl content and chloroplasts of similar structure, consistent with photosynthetic function. Moreover, those fruit chloroplasts have relatively thicker and wider grana stacks of thylakoid membranes, suggesting an adaptation to the weak light microenvironment in the fruit interior (Lichtenthaler and Burkart, 1999).

A drawback to the presence of stomata on the fruit surface is that they provide a route for loss of respiratory CO_2_. Interestingly, the parenchyma cells in the exocarp of cucumber fruits are compactly arranged with very narrow intercellular spaces (Fig. 2E). This anatomical feature evidently inhibits escape of CO_2_ generated from fruit internal respiration, but at the same time inhibits CO_2_ fixation from the atmosphere. Additionally, the guard cells on the surface are covered with a thick layer of epicuticular wax (Fig. 2B), which perhaps affects stomatal opening and closure, making them less sensitive to external factors such as light (Fig. 7A) and humidity. These structural features of the fruits minimize respiratory loss by allowing photosynthetic re-fixation of CO_2_ before it can be released to the atmosphere. This anatomical evidence suggests that internal re-fixation of respiratory CO_2_ via PEPC is quantitatively more important than direct fixation of ambient atmospheric CO_2_ by Rubisco.

### Tissue-specific expression of Rubisco and PEPC suggests that there is more than one pathway of CO_2_ assimilation

Rubisco and PEPC, two key photosynthetic enzymes, display different tissue-specific expression patterns in cucumber, which, it must be recalled, is a C_3_ plant. Rubisco is highly expressed in the palisade cells in leaves and exocarp in fruits, and to a lesser extent in vascular bundles and placenta tissue in fruits. Furthermore, analysis of relative gene expression data indicates that other important rate-limiting enzymes involved in the Calvin cycle, including fructose-1,6-bisphosphatase (FBPase) and sedoheptulose-1,7-bisphosphatase (SBPase), are present in the exocarp (Supplementary Fig. S9A). Interestingly, and similarly, other key genes involved in photosynthetic electron transport, for example light-harvesting complexes (Lhca) of PSI and light-harvesting proteins (Lhcb) of PSII also exist in the exocarp (Supplementary Fig. S9B). These results suggest that CO_2_ assimilation indeed exists in cucumber fruit, although there is much less C_3_ photosynthesis in the exocarp of ‘BYS’ compared with that of ‘ZN16’.

However, PEPC, a key enzyme in C_4_ and Crassulacean acid metabolism (CAM) photosynthetic pathways, is almost ubiquitous, and expressed abundantly in the inner tissue of cucumber fruit, especially in placenta tissue and the vascular system. A similar phenomenon has also been noted in tomato fruits in that the PEPC gene is expressed in locular tissue and the pericarp (Guillet *et al.*, 2002; Lemaire-Chamley *et al.*, 2005). PEPC is also involved in the elongation of the cotton fiber (Li *et al.*, 2010). It is possible that the main function of PEPC is to synthesize organic acids to provide the turgor pressure necessary for cell expansion during the rapid growth phase (Guillet *et al.*, 2002). Note that, as cucumber fruits grow, expression of *Csppc*, carboxylation activity of PEPC (Fig. 3), and total organic acid content (Supplementary Fig. S4) increase concurrently. It is likely that rapid fruit enlargement, at 3–9 DAA, is closely related to high organic acid accumulation. By this scenario, respiratory CO_2_ is captured by PEPC to form 4-carbon organic acids and these organic acids are then decarboxylated in green fleshy fruit. Vegetative organs, such as petioles and stems of tobacco (Hibberd and Quick, 2002) and the mid-vein of Arabidopsis (Brown *et al.*, 2010), have photosynthetic Chl-rich cells in and around the vascular bundles that possess high activities of three decarboxylation enzymes, NADP-malic enzyme (NADP-ME), NAD-malic enzyme (NAD-ME), and phosphoenolpyruvate carboxykinase (PEPCK). The CO_2_ from decarboxylation of organic acids is then converted into sucrose and starch via C_3_ photosynthesis. More experimental evidence is needed to show how the C_4_ organic acids formed by PEPC in fruits are then decarboxylated with subsequent re-fixation of the CO_2_ by Rubisco (i.e. a possible C_4_ pathway in cucumber fruit).

Although PEPC is thought to be a cytosolic enzyme (Chollet *et al.*, 1996), in cucumber fruits PEPC was located in the cytoplasm, chloroplasts, and mitochondria (Fig. 5D). This is similar to recent results demonstrating PEPC location in chloroplasts of C_3_ rice leaves (Masumoto *et al.*, 2010) and C_4_*Echinochloa glabrescens*, a paddy species (Covshoff *et al.*, 2016), and in mitochondria in castor oil seed (O’Leary *et al.*, 2011; Park *et al.*, 2012). These studies suggest that the chloroplast PEPC isoenzyme (Masumoto *et al.*, 2010; Covshoff *et al.*, 2016) and mitochondrial-associated PEPC (Park *et al.* 2012) are likely to support ammonium assimilation and anaplerotic reactions, respectively, from intermediates of the tricarboxylic acid (TCA) cycle.

In our study, in contrast to Rubisco, *Csppc* mRNA levels and PEPC activities in cucumber fruit were up-regulated in the dark (Fig. 3). It is likely that PEPC in darkened cucumber fruit underwent transcriptional expression regulation in a manner similar to the diurnal changes occurring in leaves of tobacco, a C_3_ plant: high at the end of the night and decreasing markedly during the light period (Scheible *et al.*, 2000). This result is also similar to previous observations that the activity of C_3_-specific PEPC in rice leaves is up-regulated in the dark (Fukayama *et al.*, 2003). Furthermore, recent studies found that subcellular chloroplastic and cytosolic PEPCs in rice leaves have different enzymatic properties and are probably diurnally and nocturnally regulated, respectively (Fukayama *et al.*, 2014). At present, little is known about the regulation of fruit PEPC in C_3_ plants, a subject worth further study.

### Photosynthetic contribution to fruit C accumulation

Darkening photosynthetic organs with black material is a common method to assess the relative contribution of C_3_ photosynthesis to yield (Hu *et al.*, 2012). Results of darkening experiments revealed that, under normal conditions, photosynthesis of cucumber (‘ZN16’) fruit from the C_3_ pathway of fixation of atmospheric CO_2_ contributes 9.4% of the total carbohydrate required for its own growth but that, depending on the leaf-to-fruit ratio (the lower the ratio, the larger the contribution), this figure ranges from 2.4% to 22.1% (Fig. 8). In an earlier report, the contribution to the carbon requirement of fruit under normal conditions was estimated at 1–5% for cucumber (Marcelis and Baan Hofman-Eijer, 1995). In contrast to the results on ‘ZN16’, darkening ‘BYS’ (light green fruit) with eight leaves/two fruits or four leaves/two fruits had no obvious effect on fruit yield (Supplementary Fig. S10) probably due to a lower content of photosynthesis pigments (Fig. 1), low gene expression of Rubisco, FBPase, and SBPase, and low carboxylation activity in exocarp (Fig. 3; Supplementary Fig. S9), as well as stronger respiration (Fig. 7A). Reducing the leaf proportion to two leaves/two fruits is most likely to cause ovary/fruit abortion (Supplementary Fig. S10).

 Darkening fruits decreased their source function significantly, which could lead to an increased reliance on leaf photosynthesis. Our results show that the CO_2_ assimilation rate of leaves adjacent to darkened fruits in ‘ZN16’ was relatively higher than that next to non-darkened fruits (Supplementary Fig. S11); this may compensate partly for a lack of fruit C assimilation at the level of the organ itself (Fig. 8), which was similar to the finding of a proportional increase in tomato fruit yield (Araújo *et al.*, 2011). These results also revealed complex interactions between leaves and non-foliar photosynthetic organs.

For non-foliar photosynthesis, internal CO_2_ re-fixation is also regarded as an important strategy of C acquisition (Aschan and Pfanz, 2003). Cucumber as a non-climacteric fruit maintains high respiration when mature (Paul *et al.*, 2012, Fig. 7B, C). Respiratory CO_2_ may be recaptured by PEPC, resulting in an increase in organic acid content during the rapid fruit enlargement period. However, to date, few studies have quantified the importance of internal recycling of respiratory CO_2_ in fruit. Zotz *et al.* (2003) estimated that this process reduced C losses in mature fruits of monocotyledonous epiphytic orchids, a kind of capsule with large numbers of seeds, reaching up to 60%, depending on the species. In contrast, under normal conditions, fruit photosynthesis in tomato green berry is unimportant in primary metabolism but plays a considerable role in the initiation of normal programs of seed formation (Lytovchenko *et al.*, 2011). Our data indicate that cucumber fruits grow quickly, with a C accumulation rate of 35.67 mg C fruit^–1^ h^–1^ (*n*=15) (from Supplementary Fig. S2B) during the fruit rapid expansion period. Accordingly, 7.24 µmol m^–2^ s^–1^ (equal to 0.153 µmol fruit^–1^ s^–1^, from Fig. 7C) from respiratory CO_2_, amounting to 6.61 mg C fruit^–1^ h^–1^ from deep in the fruit is recaptured or re-fixed, contributing to total C acquisition of 18.5%. According to the calculation, 88% of respiratory CO_2_ is recaptured (Fig. 7C), thus we demonstrate that the internal recycling of respiratory CO_2_ effectively reduces C losses in cucumber fruit. To our knowledge, these are the first quantitative data on *in vivo* recapture of respiratory CO_2_ in non-climacteric fleshy fruits.

In conclusion, a cucumber fruit is both a sink organ and a source organ, and its photosynthesis is characterized by both assimilating atmospheric CO_2_ via Rubisco and recapturing internal respiratory CO_2_ via PEPC. Considering both CO_2_ assimilated in the exocarp and captured and re-fixed inside, the contributions of these two aspects of fruit photosynthesis to C accumulation is 9.4% and 18.5%, respectively. These results reveal that fruit photosynthesis minimizes C losses and plays an important role in cucumber fruit growth.

## Supplementary Material

Supplementary Data
