# Supplementary material for: The complex character of photosynthesis in cucumber fruit
Source: J Exp Bot. 2017 Mar 27;68(7):1625–37. doi: 10.1093/jxb/erx034 (PMC5441898; doi:10.1093/jxb/erx034)
Supplement: Supplementary Data [file erx034_Supplementary_Data.zip › supplementary_figures_S1_S12_Table_S1.pdf]

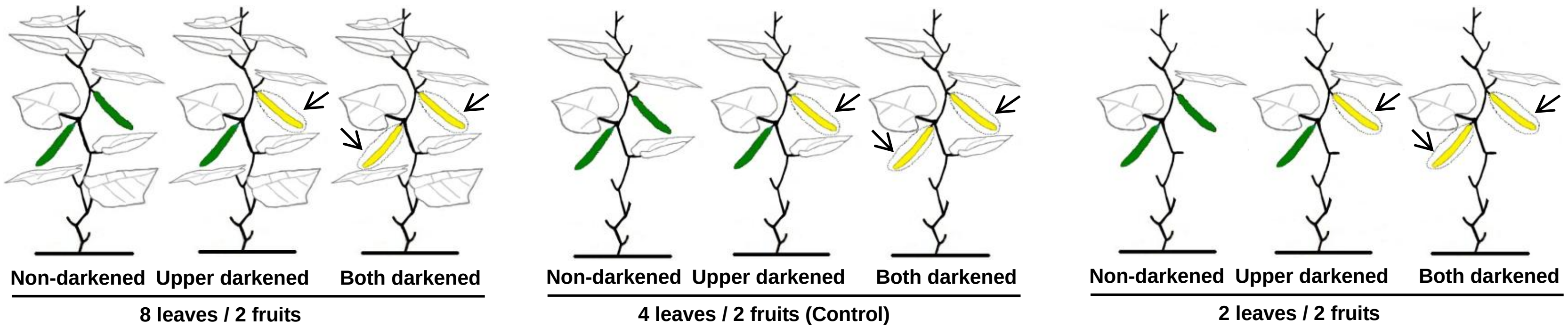

Fig. S1. A schematic diagram of darkening treatment of cucumber fruits at different leaf-to-fruit ratio. Arrows indicate upper and/or lower darkened fruits.

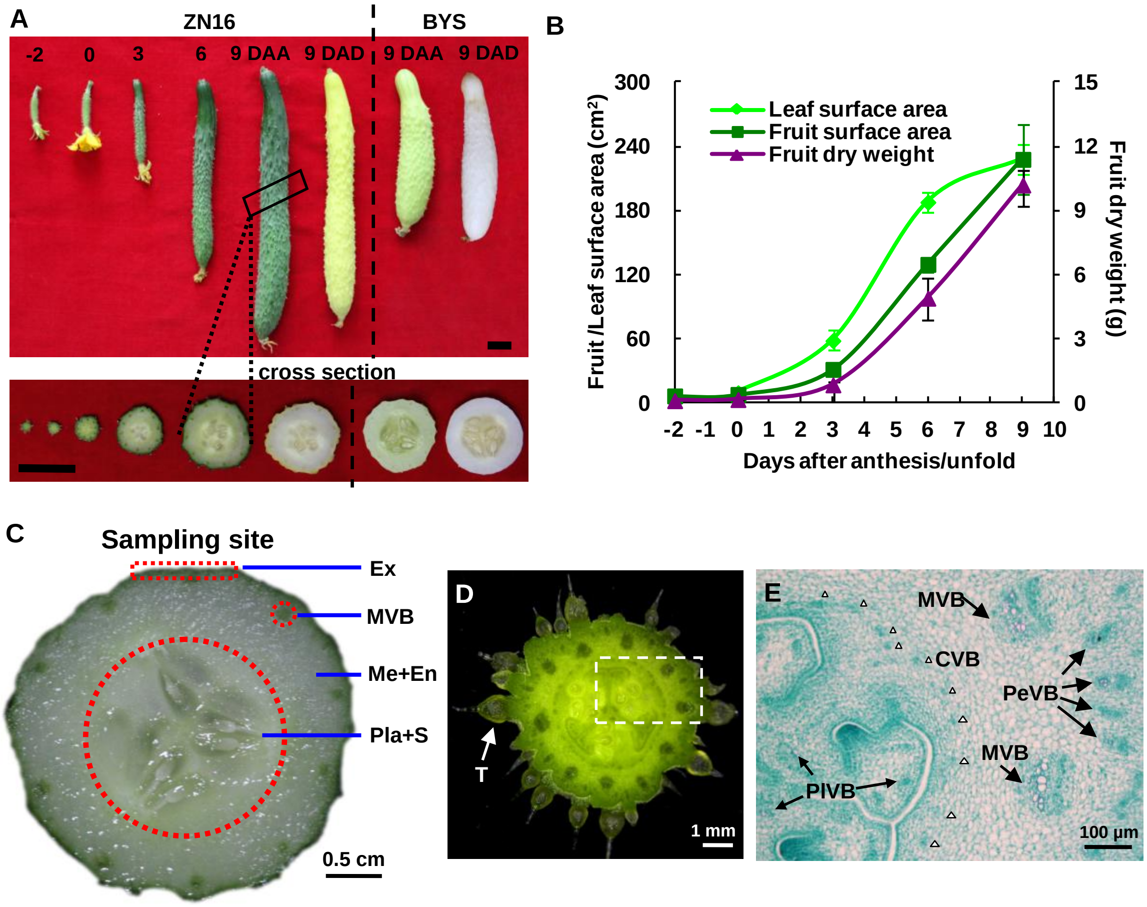

Fig. S2. The development course and microstructure of cucumber fruits. **(A)** The development course of an intact fruit and its cross sections of cucumber. 'BYS' only for 9 DAA and 9 DAD. Unless otherwise specified, segments used for analysis are the central area of fruits. Bars = 2 cm. **(B)** Growth curve of the superficial area and fresh weight of 'ZN16' fruit. Leaves and fruits were sampled on -2 (only fruit), 0, 3, 6 and 9 DAU or DAA (indicated by arrows), respectively. Error bars represent SD, n=15. **(C-E)** Cross sections of ovaries/fruits at 9 DAA **(C)** or -2 DAA **(D, E)**. **(E)** The close-up images of boxed regions in **(D)**. White triangles in **(E)** indicate CVB. **(C, D)** free-hand sections, **(E)** paraffin section. CVB, carpel vascular bundle; DAA, days after anthesis; DAD, days after darkening; DAU, days after unfolding; En, endocarp; Ex, exocarp; Me, mesocarp; PeVB, periphery vascular bundle; Pla, placenta; PlVB, placenta vascular bundle; S, seeds; T, trichome; MVB, main vascular bundle.

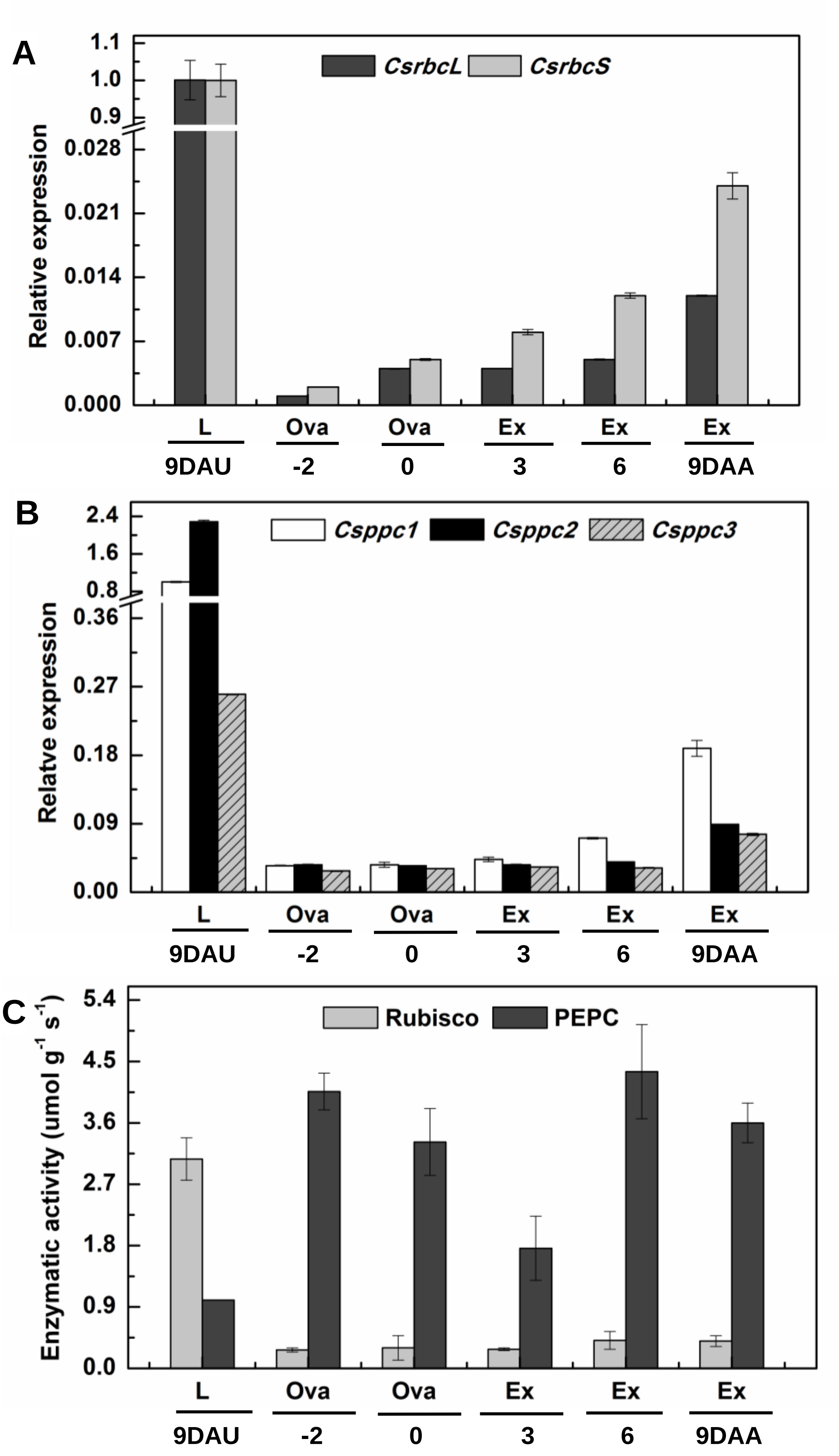

Fig. S3. Expression and activities of Rubisco and PEPC in leaves and fruits of cucumber 'BYS'. (A-B) Quantitative real-time PCR analysis of *rbcL* and *rbcS* (A) and *ppc* (B) mRNA levels. (C) Enzymatic activities of Rubisco and PEPC, which were calculated per protein concentration. Error bars represent SD, n=3. Ex, exocarp; L, leaf; Ova, ovary.

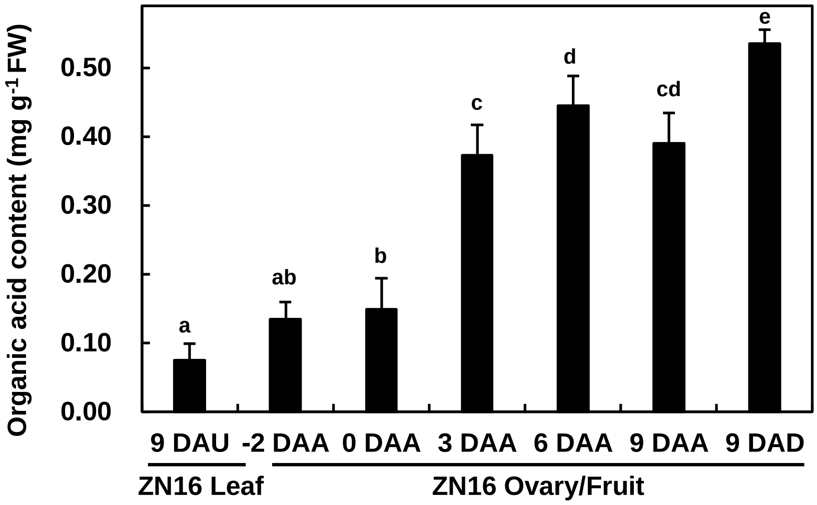

Fig. S4. Organic acid content in cucumber fruit. Means followed by different letters indicate statistically significant differences according to Tukey's test ($P < 0.05$) (n=3). DAA, days after anthesis; DAD, days after darkening; DAU, days after unfolding.

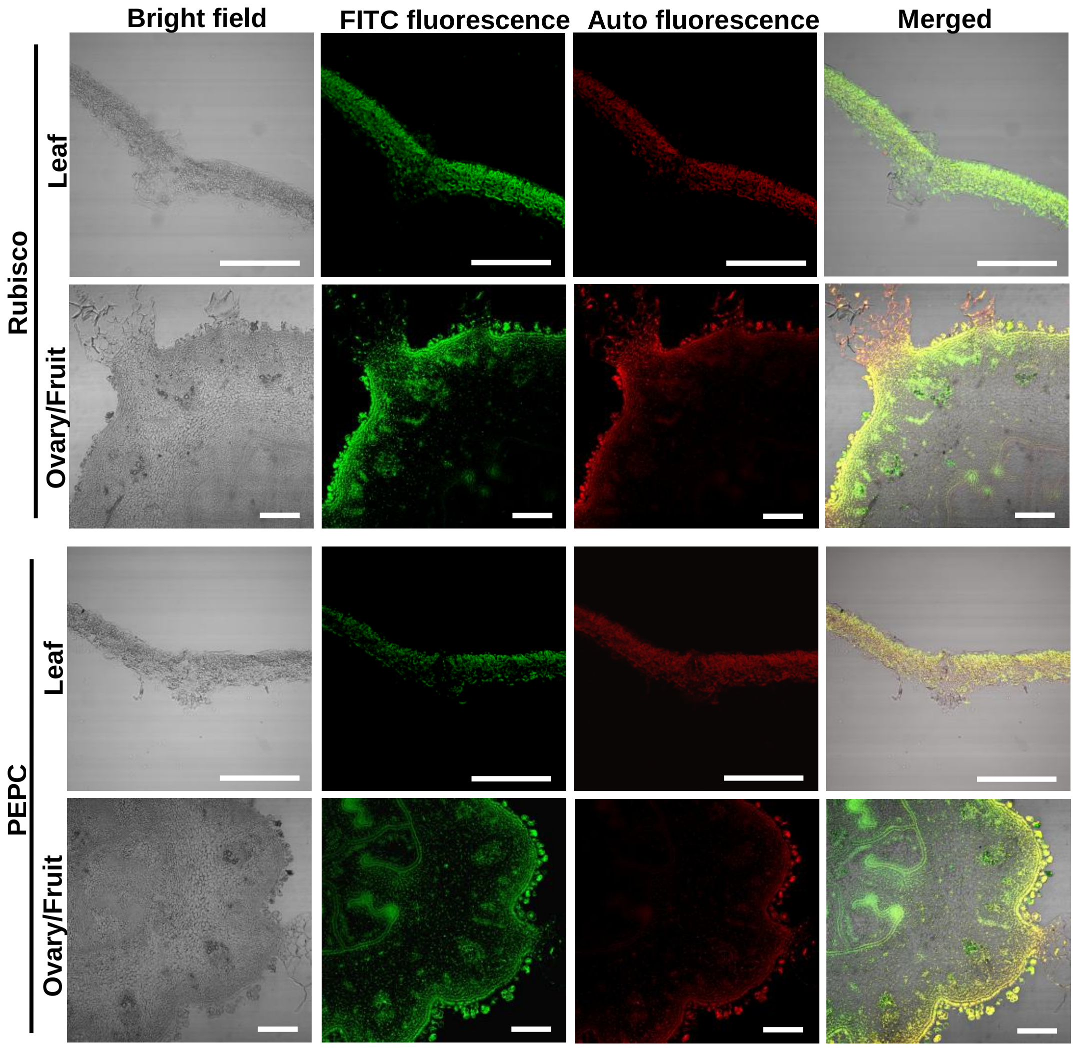

Fig. S5. Fluorescence immunohistochemical localization of Rubisco and PEPC in cucumber fruits. The cross sections of Rubisco and PEPC in leaves (0-1 DAU) and young ovaries/fruits (-2-0 DAA), respectively. The secondary antibody was fluorescein isothiocyanate (FITC) labeled anti-rabbit IgG. Green color indicates fluorescence from FITC, and red indicates auto fluorescence from chlorophyll in the chloroplasts. Bars = 200 µm.

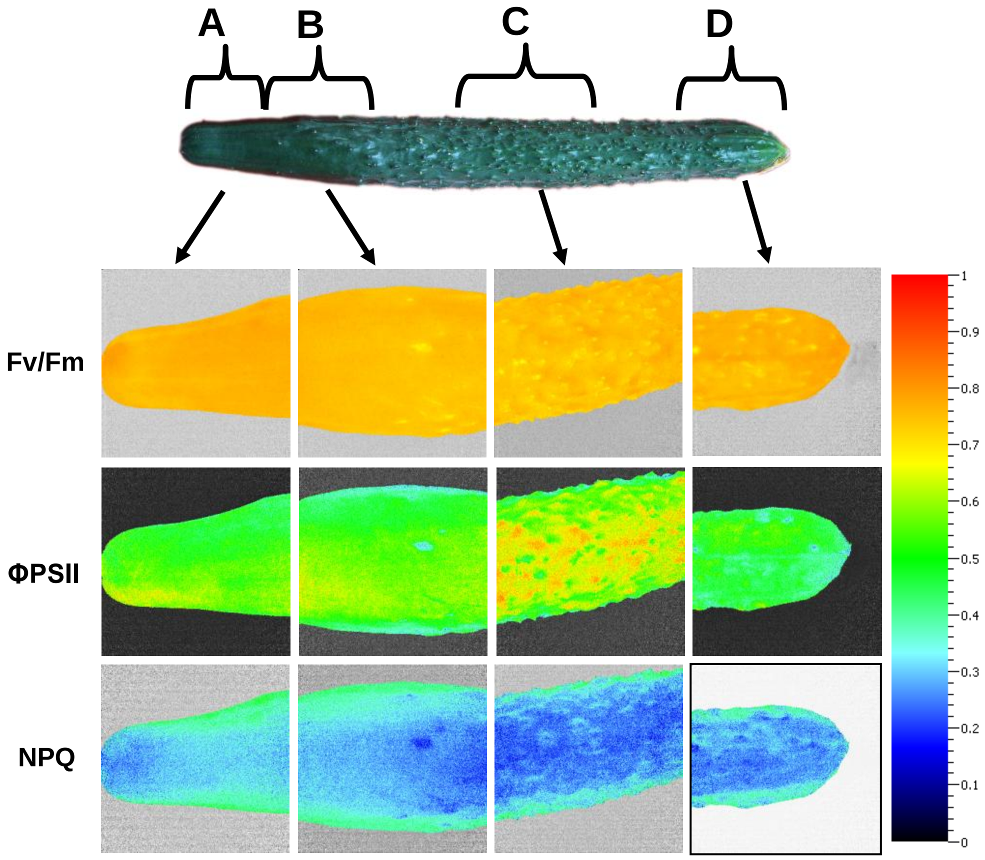

Fig. S6. Spatial difference of chlorophyll fluorescence images in mature fruit of cucumber. An intact fruit of cucumber 'ZN16' at 9 DAA, showing the sampling locations (A-D). (C) represents the main sampling area for chlorophyll fluorescence imaging at 9 DAA, as indicated in Figure 6 images. All images are normalized to the false color bar (see right column). Fv/Fm, ΦPSΠ and NPQ, see Figure 6.

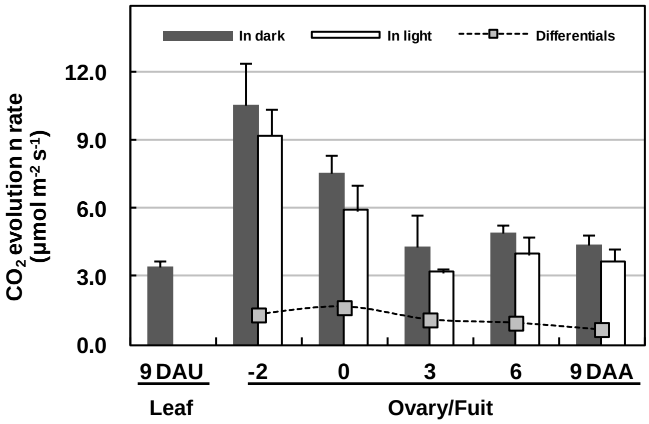

Fig. S7. CO_2 evolution rate of exocarp (peels) in dark and illuminated conditions during 'BYS' fruit development. The photon flux density (PFD) was 1000μmol m^{-2} s^{-1}. The net photosynthetic rate per unit fruit surface area (gray squares) was the difference between the CO_2 evolution rates in the light and dark. All data above were determined at ambient CO_2 between 390 and 410 mbar and at air temperature of 28℃.

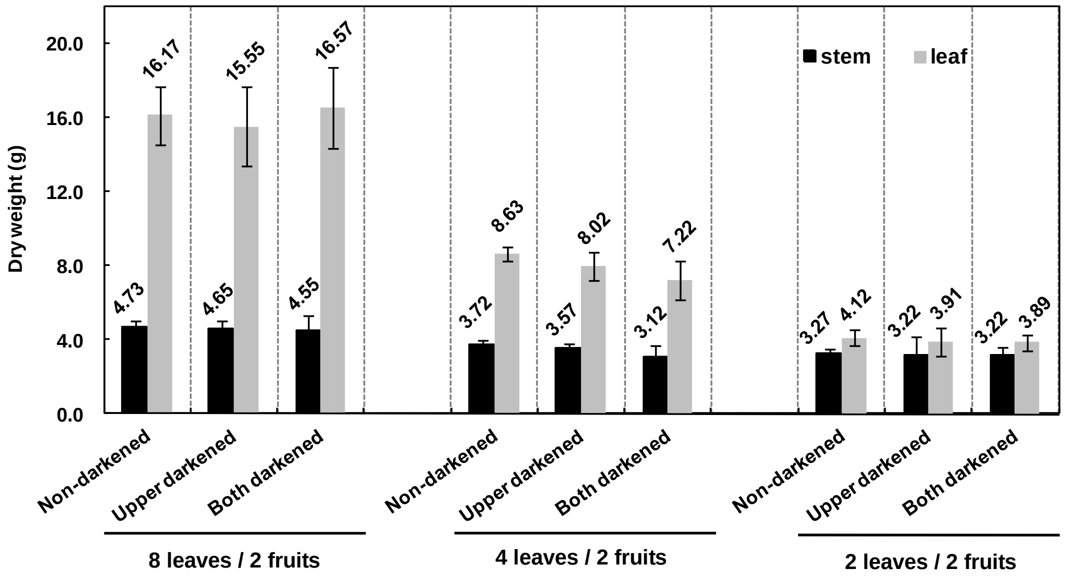

Fig. S8. Effect of fruit darkening on stem and leaf dry weight per plant of cucumber ‘ZN16’. The treatment of darkening fruits see Supporting Information Figure S1. Means ± standard deviation (SD) are given (n=4).

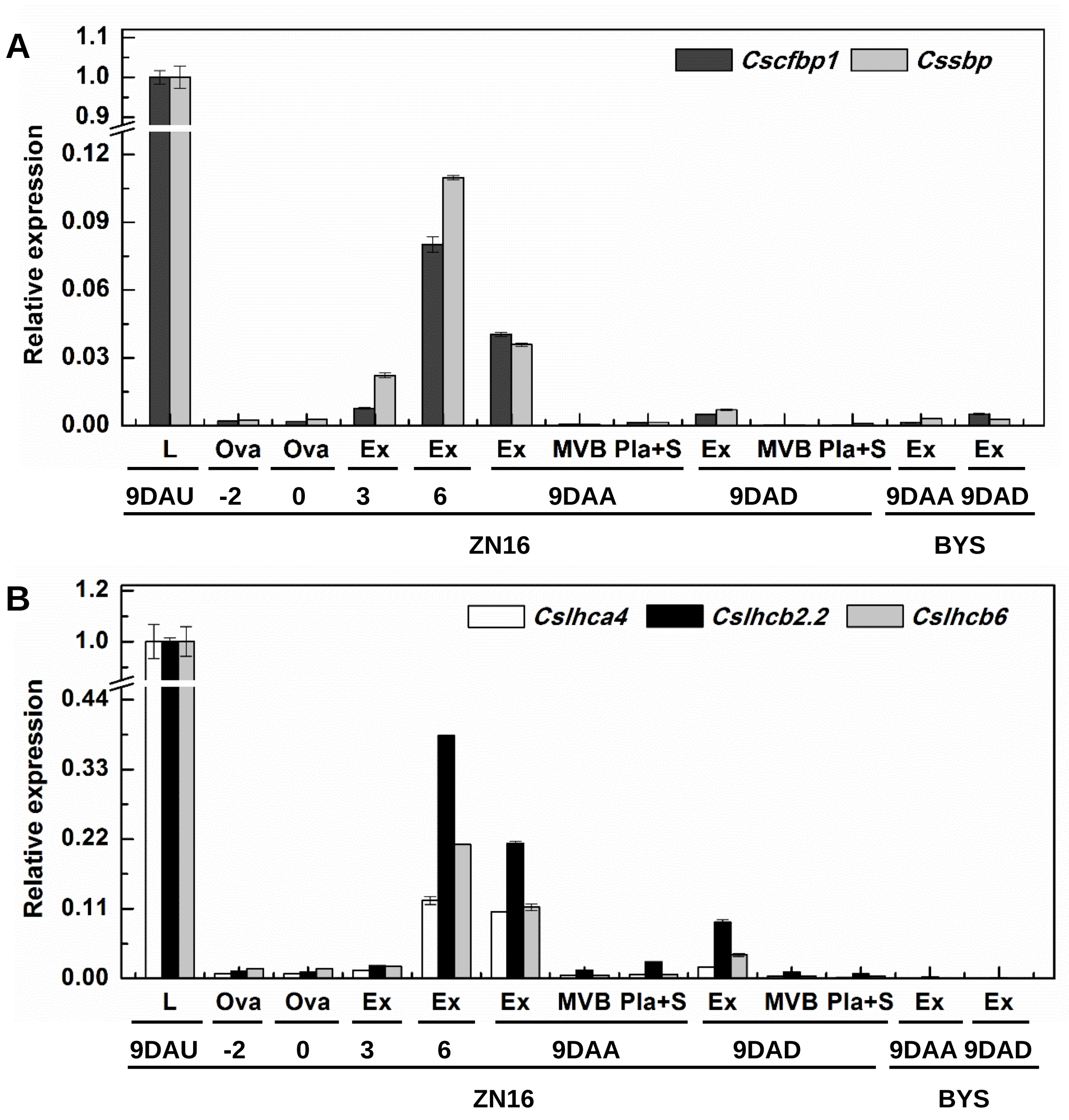

Fig. S9. Relative expression of FBPase and SBPase of the Calvin cycle (A) and key genes involved in photosynthetic electron transport (B) in cucumber fruits. Quantitative real-time PCR analysis of *cfbp1* and *sbp* (A), *lhca4*, *lhcb2.2* and *lhcb6* (B) mRNA levels. FBPase, Fructose-1,6-bisphosphatase; SBPase, Sedoheptulose-1, 7-bisphosphatase. Lhca, light-harvesting complexes of photosystem Ⅰ; Lhcb, light-harvesting proteins of photosystem Ⅱ. Error bars represent SD, n=3. Ex, exocarp; L, leaf; Ova, ovary; Pla, placenta; S, seeds; MVB, main vascular bundle.

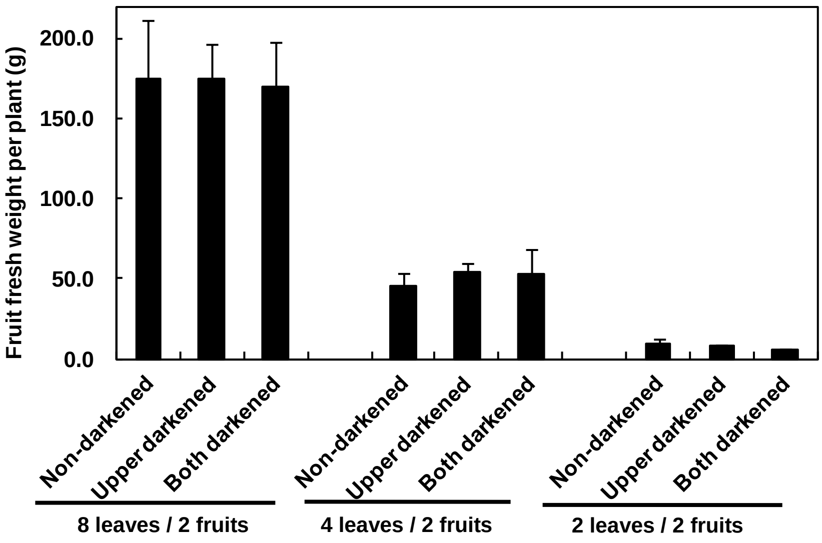

Fig. S10. Effect of darkening fruits of cucumber ‘BYS’ on fruit yield. For treatment of darkening fruits see Supporting Information Fig. S1.

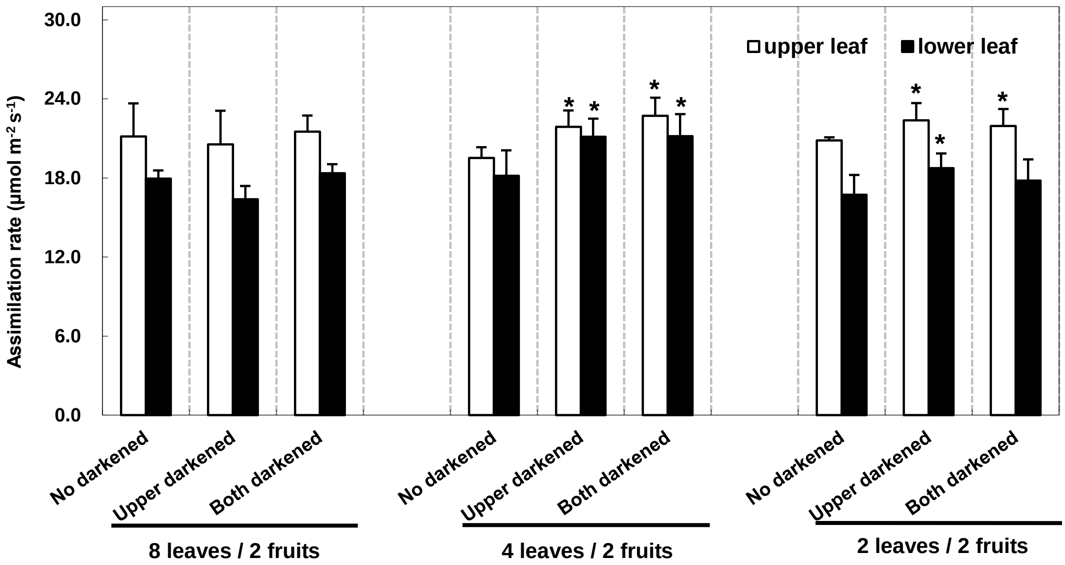

Fig. S11. Effect of fruit darkening on leaf carbon assimilation in cucumber ‘ZN16’. The treatment of darkening fruits see Supporting Information Fig. S1. Gas-exchange measurements were performed at 6-7 days after darkening treatment. Error bars represent SD, n=3. *: differ significantly from the no darkening treatment by a Student's *t*-test at $P < 0.05$ in the same leaf-to-fruit ratio.

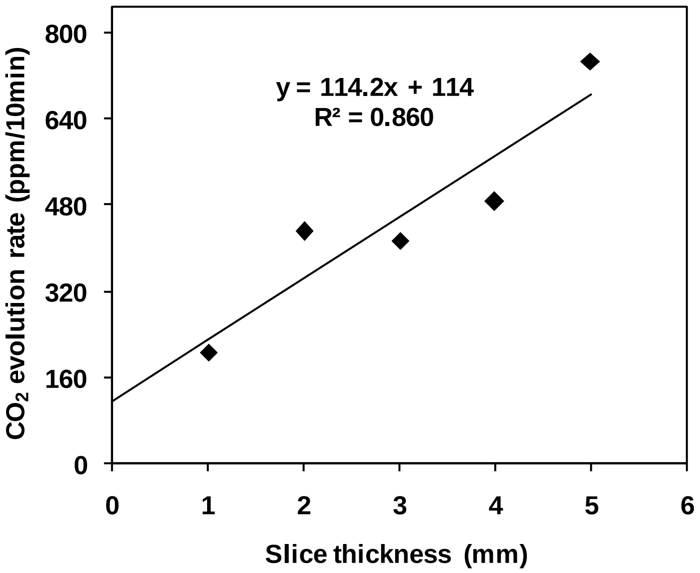

Fig. S12. The correction of wounded respiration of cucumber fruit slices caused by cutting. The intercept on the Y axis is approximately equal the wounded respiration.

Table S1. Primers used in this study.

Purpose	Direction	Primer sequence (5' to 3')	Product size (bp)	Accession number[a,b]
**Quantitative real-time PCR analysis**				
*rbc*L	F	TGACTACTTAACAGGTGGAT	185	AF206755[b]
	R	CAGCGTGAATATGGTCTC		
*rbc*S	F	TTCTTACCTGCCTGATATGA	237	Csa001020[a]
	R	TATTCCTTCTTAGCCTCCTC		
Csppc1	F	AATACCGTTCCATTGTCTTC	178	Csa015517[a]
	R	AATCTCGTCTGTGTCCAT		
Csppc2	F	GGTAAGCAAGAAGTTATGATT	199	Csa003328[a]
	R	GAGATAATATAGCAAGATGAGTAG		
Csppc3	F	TGTGGATGACTTGAGGAA	129	Csa016922[a]
	R	TTGCCAGAATCAGAATAACC		
Cslhca4	F	AACGTGTGCTCTCGGTTAAG	123	XM_004138123[b]
	R	CGGGAAGACTGCCGTTTAAATA		
Cslhcb2.2	F	CAGTTAAGAGTGCTCCACAGAG	98	XM_004144495[b]
	R	CCAGTCAGGTAAGATGGTGTTT		
Cslhcb6	F	ATGGCTTGATGGCTCACTT	101	XM_004140925
	R	GGATCAGCTCTGCTTCTCTATAC		
Cscfbp1	F	CTAGGCACAACAGAACAAAGATG	113	XM_004147851[b]
	R	CCTATGGTGAGCACGAAGATTA		
Cssbp	F	GAGTTTACGGTCCTCGCACA	95	NM_001280729[b]
	R	ATGTTGCCATTTGCCTTCGT		
Tubulin	F	GCGTTTGTCTGTTGACTATG	232	AJ715498[b]
	R	GGATACAAGACGGTTGAGG		
**In situ hybridization**				
*rbc*L	F	GAGCAGTTTATGAATGTCTACGC	551	AF206755[b]
	R	AGAGACCCAATCTTGAGTGAAATA		
*rbc*S	F	ATTCTCTCATCCGCCGC	449	Csa001020[a]
	R	GGGTATTCCTTCTTAGCCTCC		
Csppc2	F	TTATGTTTGAGTTATCTATGTGG	369	Csa003328[a]
	R	GTGAAAGCCCAAATGTAGAA		

a, b, the accession number is from cucumber genome database (http://cucumber.genomics.org.cn/) (a) or NCBI (http://www.ncbi.nlm.nih.gov/) (b). F, forward direction; R, reverse direction.
